# Association of parent-child interactions with parental psychological distress and resilience during the COVID-19 pandemic

**DOI:** 10.3389/fped.2023.1150216

**Published:** 2023-06-23

**Authors:** Mana Mann, David Harary, Shirley Louis, Tao Wang, Karen Bonuck, Carmen R. Isasi, Maureen J. Charron, Mamta Fuloria

**Affiliations:** ^1^ Department of Pediatrics, Flushing Hospital Medical Center, Queens, NY, United States; ^2^ Department of Pediatrics, Division of Neonatology, Children’s Hospital at Montefiore, Albert Einstein College of Medicine, Bronx, NY, United States; ^3^ Department of Pediatrics, Division of Neonatology, Children’s Hospital of Michigan, Detroit, MI, United States; ^4^ Department of Epidemiology and Population Health, Albert Einstein College of Medicine, Bronx, NY, United States; ^5^ Department of Family and Social Medicine, Albert Einstein College of Medicine, Bronx, NY, United States; ^6^ Department of Obstetrics & Gynecology and Women’s Health, Albert Einstein College of Medicine, Bronx, NY, United States; ^7^ Department of Pediatrics, Division of Adolescent Medicine, Albert Einstein College of Medicine, Bronx, NY, United States; ^8^ Department of Biochemistry, Albert Einstein College of Medicine, Bronx, NY, United States; ^9^ Department of Medicine, Division of Endocrinology, Albert Einstein College of Medicine, Bronx, NY, United States

**Keywords:** COVID-19 pandemic, Bronx mother baby health study, parental resilience, psychological distress, parent-child interactions

## Abstract

**Introduction:**

The effects of psychological distress/resilience on parent-child engagement (e.g., family dinners, reading) during the COVID-19 pandemic have not been well studied. Among very young children from underrepresented backgrounds enrolled in the ongoing longitudinal Bronx Mother Baby Health Study of healthy term infants, we (1) examined associations between exposures to COVID-19-related events, demographic factors and parental psychological distress and resilience; and (2) correlated these factors with parent-child engagement activities.

**Methods:**

Between June 2020-August 2021, parents of 105 Bronx Mother Baby Health Study participants aged birth-25 months completed questionnaires related to exposures to COVID-19-related events, frequency of positive parent-child engagement activities, food and housing insecurity, and parental psychological distress and resilience. Families were also asked open ended questions about the pandemic's impact.

**Results:**

29.8% and 47.6% of parents reported food and housing insecurity, respectively. Greater exposures to COVID-19-related events were associated with increased parental psychological distress. Positive parent-child interactions were associated with demographic factors and higher levels of maternal education, but not with exposures to COVID-19-related events.

**Discussion:**

This study adds to a growing body of literature on the negative impacts of COVID-19 exposures and psychosocial stressors on families during the pandemic, supporting the need for enhanced mental health resources and social supports for families.

## Introduction

COVID-19 associated effects on health, educational, economic, and social sectors have impacted children, families, and communities worldwide. New York City (NYC) was among the first metropolitan areas to experience the pandemic in the United States. The Bronx, in particular, reported the most severe COVID-19-related outcomes, with the highest hospitalization and death rates in NYC (1). Unemployment in the Bronx peaked at nearly 25% in May 2020, further devastating NYC communities with the highest pre-pandemic rates of poverty and food insecurity (2).

Worldwide closures of daycares, schools and recreation centers resulted in an estimated 1.38 billion children shifting to remote learning (3, 4). Many families experienced challenges with remote schooling, childcare, and professional obligations due to limited community- and family-level resources (5). Increased stress and adverse mental health effects were reported among parents (6, 7), both during pregnancy and in the postpartum period (8–10). Hospital policies during the pandemic often forced pregnant women to give birth unaccompanied by their partners which further heightened psychological distress (10).

The pandemic also worsened food and housing insecurity and poverty which particularly affect young children who are highly dependent on their primary caregivers for their physical and emotional needs (11). Although studies have found that young children's exposure to early life stress impacts their development and overall health (12, 13), parents can buffer these negative effects by externally regulating their children's emotions (14) and supporting development of their intrinsic capacity for self-regulation (15). Positive parent-child interactions and engagement activities mitigate some of these negative factors and promote children's socioemotional development (16–19) and resiliency to stress (19, 20).

Postpartum mental health symptoms during the pandemic may negatively impact maternal-infant bonding (21, 22). Recent studies have shown that parents of children 10 months–17 years of age (mean age 8.84 years) more effectively buffer the negative effects of pandemic-related stress by supporting their children through negative emotions and more consistently maintaining household routines (23). Others have reported that lower quality parenting during the COVID-19 pandemic was associated with multiple household and pandemic risk factors, with caregiver depression linked to parent-child relationship disruptions among parents of children 1.5–8 years of age (24). However, few studies have examined parental psychological distress, resilience and regular engagement in parent-child/family activities during the COVID-19 pandemic, especially among parents of very young children and communities of color (23, 24).

Investigating the impact of COVID-19-related factors on parental mental health and parent-child engagement in activities is critical to identifying, informing, and planning services, education, and policies to support young children and families in the setting of high stress or adversity. Therefore, in children and their mothers enrolled in the “Bronx Mother Baby Health Study” (Bronx MomBa Health Study), an ongoing longitudinal study of epigenetic mechanisms underlying intrauterine growth restriction (IUGR)-mediated obesity from birth to two years of age, we sought to identify: (1) associations between parental psychological distress, resilience, parental perception of their child's psychological distress, exposures to COVID-19-related events, and social-demographic factors, and (2) associations between psychological distress and resilience and parent-child engagement activities. Parents were also asked open ended questions to better understand the impact of the COVID-19 pandemic on families. We hypothesized that there would be a positive association between exposures to COVID-19-related events and parental psychological distress. Furthermore, positive engagement in parent-child activities would be negatively associated with parental psychological distress and positively associated with parental resilience.

## Methods

### Study sample

The Bronx MomBa Health Study is an ongoing longitudinal study that aims to understand the pathogenesis of early childhood obesity (from birth to two years), by examining epigenetic mechanisms underlying IUGR-mediated alterations in DNA methylation of CD^3+^ T-cells*.* Infants of pregnant persons who received prenatal care and delivered at a large urban, private, non-profit hospital in the Bronx, NY were enrolled in the study. Inclusion criteria for this study include healthy term singleton pregnancy with no prenatal history of depression, smoking after the first trimester of pregnancy, or gestational/type 2 diabetes. Infants who were large for gestational age, and those with an Apgar score <7 at 5 min of age, chromosomal or congenital abnormalities, congenital infections, or inborn errors of metabolism were excluded. Infant anthropometric measurements, cord- and peripheral blood samples, and stool samples were obtained at multiple timepoints over the course of the first 2 years of life.

From 9/2018–8/2021, 377 pregnant women were approached and 217 (57.6%) consented to study participation. Fifty-five of the consented participants (25.3%) were excluded from the study for the following reasons: cord blood sample not collected, infant large for gestational age or infant admitted to the intensive care nursery.

**Figure 1:**
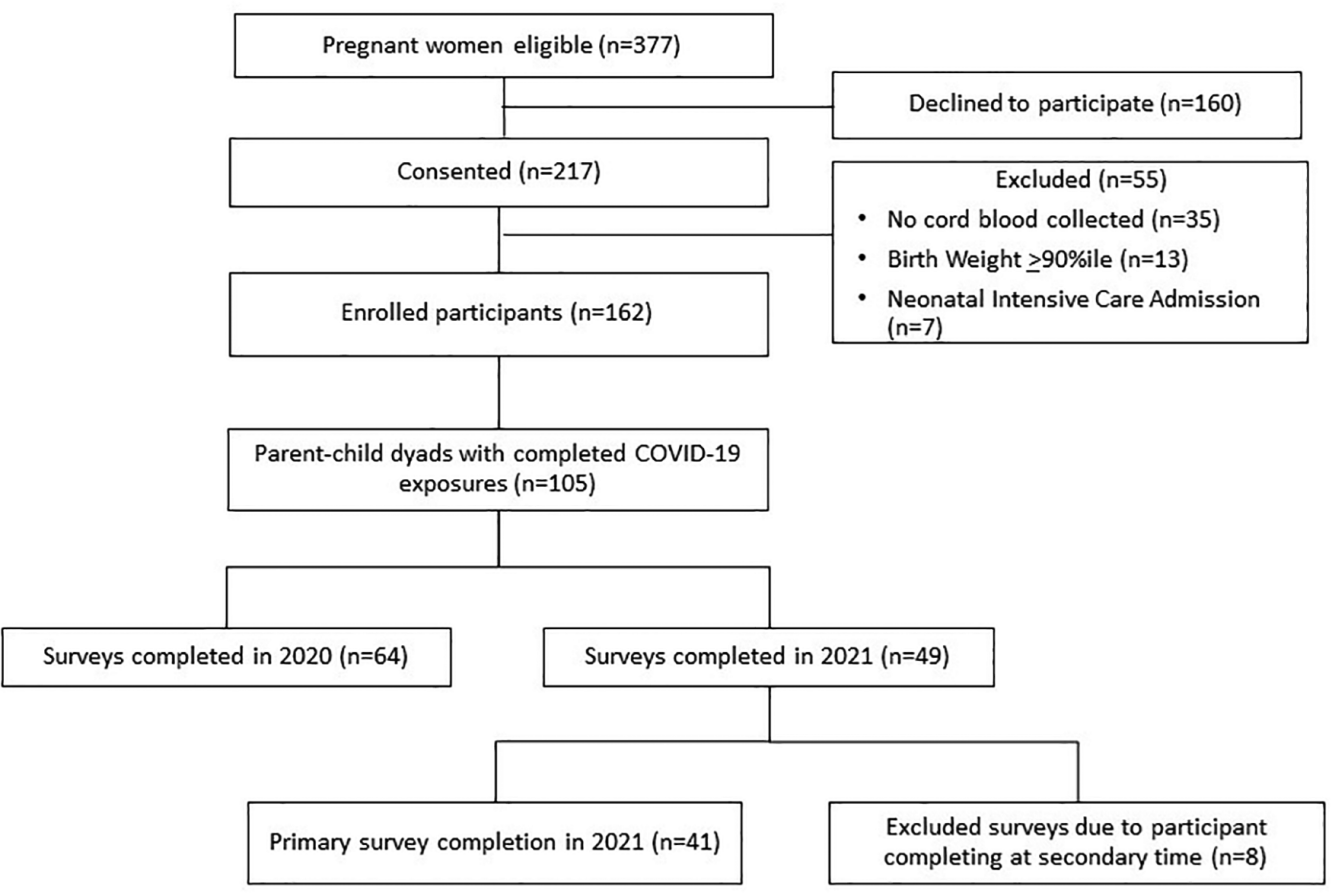
Flow diagram of study participation.

In March 2020, the study was paused for new enrollment and in person visits because of the COVID-19 pandemic. Enrollment and study visits were resumed in September 2020. During the early stages of the pandemic, the original study protocol was amended in order to collect information related to the pandemic's effects on our study cohort. A total of 105 parents whose children were scheduled for study-related visits between 6/2020 and 8/2021 participated in this amended study and provided information regarding their exposures to COVID-19-related events (Figure 1). Parents completed questionnaires regarding demographics, food and housing insecurity, psychological distress, resilience, and involvement in parent-child engagement activities.

This study was approved by the Montefiore Medical Center/Albert Einstein College of Medicine Institutional Review Board (NCT03402139).

### Study measures

Since in-person study-related visits were halted during the height of the pandemic, some of the study participants completed the questionnaires either over the telephone or mailed the completed questionnaires to the study team whereas others completed the study questionnaires during the in-person study visits once on-site study-related activities resumed in September, 2020. All study questionnaires were available in both English and Spanish.

#### Exposures to COVID-19-related events

Parents completed the COVID-19 Exposure and Family Impact Survey (CEFIS) (25), a questionnaire based on a trauma framework that aims to understand exposures as well as the impact of the COVID-19 pandemic on families and their children. Part 1 of the questionnaire focuses on COVID-19 related exposures/events, includes 25-items in six categories (designation as an essential worker, access to essentials, disruptions to living conditions, income loss and family caregiving and activities) with a yes/no answer. A higher Part 1 score indicates higher COVID 19-related exposures. Part 2 of the questionnaire focuses on the impact of the pandemic on families (personal well-being, family interactions, distress) and consists of 12-items, 10 of which use a 4-point Likert scale (made it a lot better; made it a little better; made it a little worse; and made it a lot worse). The last two questions in Part 2 of the questionnaire relate to COVID-19-related psychological distress experienced by the caregivers (Question 36) and their children (Question 37) and are rated on a 10-point Likert scale. Higher scores for Questions 36 and 37 indicate higher levels of psychological distress experienced by parents and their children, respectively. Part 3 of the scale includes an open-ended prompt for parents to share their positive and negative experiences during the pandemic. The COVID-19 Exposure and Family Impact Survey was registered on April 22, 2020 with the National Institutes of Health Disaster Information Management Research Center (https://www.niehs.nih.gov/research/programs/disaster/database/cefis_covid_questionnaire_english_42220_final_508.pdf) and made widely available to the public. Here, we report on the results from Part 1 of the survey, questions 36 and 37 from Part 2 of the survey, and Part 3 of the survey.

#### Kessler 10 psychological distress scale

Parents completed the Kessler 10 Psychological Distress Scale (K10), a questionnaire developed to yield a global measure of psychological distress based on questions related to anxiety and depressive symptoms in the most recent 4-week period (26). Each item is scored on a 5-point scale from 1 (none of the time) to 5 (all of the time), with a total sum ranging from 10 to 50. As few studies have collected normative data of psychological distress among a diverse group of parents of very young children (including postpartum mothers) (27), we elected to analyze psychological distress as a continuous outcome, with higher scores indicating greater levels of distress. This analytical strategy is consistent with Kessler's recommendations against simple summing of items and use of cutoff scores (26).

#### Parental resilience

The 14-item Resilience Scale (RS14) is a validated, self-report scale evaluating the main characteristics of resilience and the ability to overcome adversity (28). Each item is scored on a 7-point scale from 1 (strongly disagree) to 7 (strongly agree) with a total sum ranging from 14 to 98; higher scores indicate higher resilience. Previous studies have shown good reliability of the scale in adults (29–33).

#### Parent-child engagement activities

Parents reported on the frequency of positive engagement activities with their child including telling stories, playing games, and singing as well as household routines for meals and bedtime. These questions were based on national surveys from the Early Childhood Longitudinal Study, Birth Cohort which have been utilized previously by others (17). Activities were categorized and analyzed independently as present if children and caregivers engaged in: (1) family dinners ≥ 5 days/week, (2) sleep routines ≥ 5 days/week, (3) reading ≥ 3 times/week, (4) storytelling ≥ 3 times/week, (5) singing ≥ 3 times/week, or (6) playing ≥ 3 times/week (17).

#### Food and housing insecurity

Food insecurity was assessed by parent report of a two-item screening questionnaire from the U.S. Department of Agriculture 18-item food security screening measure (34). The questions include: (1) “We worried whether our food would run out before we got money to buy more” and (2) “The food we bought just didn’t last and we didn’t have money to get more” (34). Each item was scored on a 3-point scale (often true; sometimes true; never true; don't know). Food insecurity was categorized as present if parents reported either “Sometimes True” or “Often True” for either of the two questions. We also asked the following question: “Did your household participate in a food assistance program?” This question was scored on a 4-point scale (often true; sometimes true; never true; don't know). Household participation in a food assistance program was categorized as present if parents reported either “Sometimes True” or “Often True” for this question.

Housing insecurity was determined using the following question: (1) “Did you worry there would not be enough money to pay for rent?” (35). This item was scored on a 3-point scale (often true; sometimes true; never true; don't know). Housing insecurity was categorized as present if parents reported either “Sometimes True” or “Often True” for this question.

### Other patient characteristics

Child characteristics included infant sex, race, and ethnicity. Ethnicity was categorized as Hispanic/Latino if either parent self-reported as being Hispanic/Latino. If multiple races were selected or if parents refused to self-identify as belonging to any race, this variable was categorized as “other.” Parent characteristics included language of survey completion and educational attainment.

### Statistical analysis

All analyses were performed using R package (https://www.r-project.org/; version 4.1.2) and SPSS version 29. This study has a sample size of 105 for our first study objectives of identifying associations between parental psychological distress and resilience, with exposures to COVID-19-related events, family- and parent-level factors and 58–72 for our second study objectives of identifying associations between these factors and parent-child engagement activities, which provides 80% power at significance level of 0.05 to detect a moderate correlation of *r* = 0.27 and *r* = 0.33–0.36, respectively. Spearman's correlation was used to examine associations between COVID-19-related events, psychological distress and resilience and between psychological distress and resilience and parent-child engagement activities. Associations between maternal characteristics and psychological distress and resilience were analyzed using Spearman's correlation, *T*-test or ANOVA as appropriate. Associations between categorical maternal characteristics and parent-child engagement activities were examined using *χ*^2^-test. We used an alpha level of 0.05 for all statistical tests. For the qualitative analysis, the content of parent responses to the open-ended COVID-19 Exposure and Family Impact Scales question were categorized under broad themes.

## Results

### Demographics of study participants

Table 1 describes characteristics of study participants. Overall, 73.3% of participants were Hispanic and 28.6% completed the surveys in Spanish; 52.4% and 31.4% were White and Black, respectively. Of the study participants, 29.4% graduated from high school, and 60.8% had more than high school education. After the pandemic start, almost one third (29.8%) of participants reported food insecurity, and almost one-half (46.9%) reported use of food assistance programs. Approximately one-half (47.6%) of our study participants reported that they were experiencing some level of housing insecurity.

There were no differences between study participants who either completed the questionnaires over the telephone or mailed the questionnaires to the study team and those who completed the questionnaires during the in-person study visit.

**Table 1:** Characteristics of study participants (*n*** = **105).

Maternal age (years; mean ± SD)	30.9 ± 5.9
Race (*N*; %)	
White	55 (52.4)
Black	33 (31.4)
Other	17 (16.2)
Ethnicity (*N*; %)	
Hispanic	77 (73.3)
Non-Hispanic	28 (26.7)
Mode of Delivery (*N*; %)	
Vaginal delivery	42 (40)
C-section	63 (60)
Infant Sex (*N*, %)	
Male	52 (49.5)
Female	53 (50.5)
Education (*N*; %)	
<High school	10 (9.8)
High school/GED	30 (29.4)
>High school	62 (60.8)
Language of Completed Survey (*N*; %)	
English	75 (71.4)
Spanish	30 (28.6)
Food Insecurity (*N*; %)	
No	40 (70.2)
Yes	17 (29.8)
Use of Food Assistance Programs (*N*; %)	
No	34 (53.1)
Yes	30 (46.9)
Housing Insecurity (*N*; %)	
No	33 (52.4)
Yes	30 (47.6)

### Exposures to COVID-19-related events

**Figure 2:**
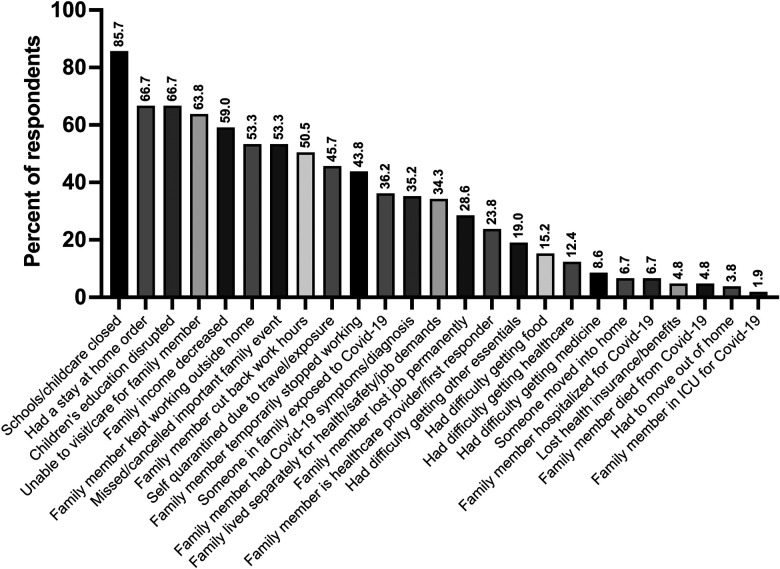
Reporting of individual items of the COVID-19 exposure and family impact scales (CEFIS), part 1 among the mother baby health study participants. (*n*=105).

The mean COVID-19 Exposure and Family Impact Survey Part 1 score in our cohort was 8.3 + 4.2. Figure 2 illustrates the percent of participants who reported each of the COVID-19 Exposure and Family Impact Survey items. Notably, 60% reported a decrease in family income; 44% that a family member had to temporarily stop working; 36% that a family member was exposed to COVID-19; 35% that a family member was symptomatic or diagnosed with COVID-19; and 29% that a family member lost their job permanently.

### Parental psychological distress

In our cohort, the value for Cronbach's Alpha for the K10 scale was *α* = .931. The mean score for psychological distress was 14.7 ± 5.8. Higher levels of psychological distress were associated with race {Black 16.5 + 6.2; White 14.6 + 6.1; Other 12.1 + 2.7; [*F*(2, 99) = 3.178, *p* = .046]}, and housing insecurity [yes: 16.0 + 6.1 vs. no: 12.9 + 3.6; *p* = .022; 95% CI (−5.73, −.46)]. There was no association between psychological distress levels and maternal age, ethnicity, education, and food insecurity. Parents who reported food insecurity were more likely to use food assistance programs [*X*^2^ (1, *N* = 57) = 9.20, *p* = .003]. Parents who reported use of food assistance programs had higher K10 scores, but this association did not reach statistical significance [yes = 15.9 + 6.3 vs. no = 13.4 + 4.1; *p* = .075; 95% CI (−5.11, .25)].

There was a positive correlation between COVID-19 related exposures (Part 1 of COVID-19 Exposure and Family Impact Survey) and parental psychological distress as measured by the K10 questionnaire [*r_s_*(102) = .26, *p* = .009], parental psychological distress as measured by Question 36 of COVID-19 Exposure and Family Impact Survey Part 2 [*r_s_*(96) = .26, *p* = .012] and parent-reported psychological distress in their children as measured by Question 37 of COVID-19 Exposure and Family Impact Survey Part 2 [*r_s_*(91) = .34, *p* = .001] (Table 2). There was a strong positive correlation between parental psychological distress as measured by Question 36 and parent-reported psychological distress in their children as measured by Question 37 of COVID-19 Exposure and Family Impact Survey Part 2 [*r_s_*(91) = .53, *p* < .001].

**Table 2:** Spearman’s correlation between COVID-19 exposure and family impact survey (CEFIS) part 1 score, resilience and psychological distress.

	CEFIS Part 1 *r_s_* (95% CI)	Resilience
Overall parental psychological distress (K10 questionnaire)	.258** (.061 −.435)	−.323** (−.500 −.120)
Parental psychological distress (CEFIS questionnaire; Q36)	.256* (.052 −.439)	−.068 (−.280 −.151)
Parent-reported psychological distress experienced by their children (CEFIS questionnaire; Q37)	.336 (.134 −.512)	−.097 (−.313 −.129)

* *p* < .05.

** *p* < .001.

### Parental resilience

In our cohort, the value for Cronbach's Alpha for the RS14 scale was *α* = .942. The mean score for resilience was 86.4 + 13.9. Maternal age, race, ethnicity, education, food and housing insecurity, and COVID-related events were not associated with resilience as measured by the RS14 questionnaire. As shown in Table 2, there was an inverse relationship between parental resilience and psychological distress as measured by the K10 questionnaire [*r_s_*(92) = −.32, *p* = .002]. There was no association between resilience and parental psychological distress and parent-perceived psychological distress in their children as measured by Questions 36 and 37 of the COVID-19 Exposure and Family Impact Survey Part 2.

### Parent-child engagement activities

Table 3 shows the association between maternal and child characteristics and parent-child engagement activities. Eating dinner as a family ≥5 times/week was associated with non-Hispanic ethnicity [*X*^2^ (1, *N* = 66) = 8.58, *p* = .003]. Regular sleep routine was associated with higher maternal education [*X*^2^ (2, *N* = 56) = 7.94*, p* = .026]. Although higher maternal education was associated with more frequent reading, this association did not reach statistical significance [*X*^2^ (2, *N* = 72) = 6.02, *p* = .052]. Completing the surveys in English was associated with reading >3 times a week [*X*^2^ (1, *N* = 72) = 4.93, *p* = .037]. Story telling was associated with female sex of the child [*X*^2^ (1, *N* = 72) = 7.80, *p* = .008].

**Table 3:** Association of maternal and child characteristics with parent-child engagement activities.

	Study population, *N* (%)	Family dinner ≥5 times/wk, *N*%; *N* = 66 (no = 18)	Regular sleep routine ≥5 times/wk, *N*%; *N* = 58, (no = 15)	Reading ≥3 times/wk, *N*%; *N* = 72 (no = 20)	Story telling ≥3 times/wk, *N*%; *N* = 72 (no = 30)	Singing ≥3 times/wk, *N*%; *N* = 69 (no = 13)	Playing ≥3 times/wk, *N*%; *N* = 71 (no = 8)
Race							
White	55 (52.4)	24 (43.6)	23 (41.8)	27 (49.1)	23 (41.8)	27 (49.1)	33
Black	33 (31.4)	16 (48.4)	11 (33.3)	15 (45.5)	10 (30.3)	18 (54.5)	19
Other	17 (16.2)	8 (47.1)	9 (52.9)	10 (58.8)	9 (52.9)	11 (64.7)	11
*p value*		*X^2^ (2, N = 66) = 1.79,* *p = .431*	*X^2^ (2, N = 58) = .58,* *p = .789*	*X^2^ (2, N = 72) = 1.26,* *p = .591*	*X^2^ (2, N = 72) = 1.95,* *p = .396*	*X^2^ (2, N = 69) = 6.114, p = .052*	*X^2^ (2, N = 71) = 3.73,* *p = .141*
Ethnicity							
Non-Hispanic	28 (26.7)	17 (60.1)	11 (39.3)	13 (46.4)	13 (46.4)	14 (50.0)	15 (53.6)
Hispanic	77 (73.3)	31 (40.3)	32 (41.6)	39 (50.6)	29 (37.7)	42 (54.5)	48 (62.3)
*p value*	* *	*X^2^ (1, N = 66) = 8.58,* ***p****** = .003***	*X^2^ (1, N = 58) = 2.42,* *p = .156*	*X^2^ (1, N = 72) = .20,* *p = .764*	*X^2^ (1, N = 72) = 3.01,* *p = .099*	*X^2^ (1, N = 69) = .02,* *p = 1.000*	*X^2^ (1, N = 71) = .00,* *p = 1.000*
Child Sex							
Female	52 (49.5)	26 (50.0)	25 (48.1)	28 (53.8)	28 (53.8)	30 (57.7)	34 (65.4)
Male	53 (50.5)	22 (41.5)	18 (40.0)	24 (45.3)	14 (26.4)	26 (49.1)	29 (54.7)
*p value*		*X^2^ (1, N = 66) = .09,* *p = .789*	*X^2^ (1, N = 58) = 1.47,* *p = .247*	*X^2^ (1, N = 72) = .08,* *P = .798*	*X^2^ (1, N = 72) = 7.80,* ***p****** = .008***	*X^2^ (1, N = 72) = .00,* *p = 1.000*	*X^2^ (1, N = 72) = .04,* *p = 1.000*
Language of Completed Survey							
English	75 (71.4)	40 (53.3)	33 (44.0)	42 (56.0)	33 (44.0)	50 (66.7)	47 (62.7)
Spanish	30 (28.6)	8 (26.7)	10 (33.3)	10 (33.3)	9 (30.0)	19 (63.3)	16 (53.3)
*p value*	* *	*X^2^ (1, N = 66) = 2.17,* *p = 0.180*	*X^2^ (1, N = 58) = .58, p = 0.502*	*X^2^ (1, N = 72) = 4.93,* ***p****** = .037***	*X^2^ (1, N = 72) = 1.27,* *p = .289*	*X^2^ (1, N = 72) = .958,* *p = .491*	*X^2^ (1, N = 71) = .531,* *p = .673*
Maternal Education							
Less than high school education	10 (9.5)	3 (30.0)	2 (20.0)	3 (30.0)	2 (20.0)	4 (40.0)	5 (50.0)
High school graduate	30 (28.6)	12 (40.0)	9 (30.0)	13 (43.3)	12 (40.0)	18 (60.0)	19 (63.3)
More than high school education	62 (59.0)	32 (51.6)	30 (48.4)	35 (56.5)	27 (43.5)	33 (53.2)	38 (61.3)
*p value*	* *	*X^2^ (2, N = 64) = 1.33, p = .599*	*X^2^ (2, N = 56) = 7.94,* ***p = 0.026***	*X^2^ (2, N = 72) = 6.02, p* * = 0.052*	*X^2^ (1, N = 72) = 4.93, p = 0.194*	*X^2^ (2, N = 72) = .02, p = 1.000*	*X^2^ (2, N = 72) = .934, p = .709*

The bold values are significant *p* values.

There was no correlation between parental psychological distress or resilience and engagement in any of the parent-child activities. However, parents with lower levels of psychological distress were more likely to engage in story telling ≥3 times/week (*p* = .08), but this association did not reach statistical significance. Additionally, parents with higher resilience scores were more likely to have family dinners ≥5 times/week, but this association did not reach statistical significance (*p* = .068).

### Positive and negative effects of COVID-19 as reported by families

Fifty-seven parents (54.2%) answered the open-ended CEFIS question regarding positive and negative effects of the pandemic. Parents reported on the economic effects of the pandemic including income/job loss, and difficulty with paying rent (*n* = 9). Parents discussed disruption to daily life, with one participant noting she was unable to bring her partner to prenatal visits. Some participants described feelings of being “locked in” during this time (*n* = 7). Others discussed concern for themselves and family members of contracting COVID-19 (*n* = 7). One mother who developed COVID-19 during pregnancy wrote:

“I have an older, preteen daughter who basically cared for herself and for me while I was pregnant and COVID positive. I don't remember how she fed or cared for herself. Having COVID while pregnant was very difficult for me with chills and fever for days. I was unable to eat or drink anything. During this time, I was a single parent of a preteen daughter and was going through pregnancy alone with a child’s support. I did not have any other physical, emotional, or economical support. After days without food, I was able to apply so that we could receive food at home. Being infected with COVID during pregnancy was very scary and I was nervous if the infection had affected my baby.”

Additional stressors described related to challenges in remote schooling, childcare, quarantining, limiting children's social and outdoor activities, navigating parental and professional responsibilities, and having little personal time. One parent reflected on their experience as:

“I have learned not to want what is more than necessary and to keep on fighting.”

Parents also shared concerns about the implications of the pandemic on the physical, psychological, and educational well-being of their children. Parents commented on their children's lack of socialization (*n* = 6), as well as behavior changes due to COVID-19-related disruptions (*n* = 4). One mother described the disruption to her family's life:

“Schools, playgrounds, and parks being closed makes finding time/place for young children to learn about the actual world around them very difficult and, frankly, depressing. We literally did virtual physical therapy in the driveway to our garage for our apartment building one day because there was literally no other outdoor space available other than the road. Thankfully it was not a busy 30 min. Children, especially young children, need room to move and explore their worlds. The fact that all the adults in their lives are masked outside of home and they regularly have gun-shaped devices pointed at their heads (to take their temperatures) is gravely disturbing to me as a parent. I have boys that love nature and need to run around and explore things.”

Notably, many participants reflected on more family time (*n* = 38). Distinction between immediate and extended family was raised, with some noting immediate family cohesiveness increased while others highlighted decreased interaction with extended family members. Some participants provided specific examples, including engaging in creative activities, mealtimes, cooking, and teaching children to be more independent. Participants also highlighted opportunities to watch their children grow and achieve milestones. Moreover, they expressed admiration for their children's resilience.

## Discussion

Families of young children are particularly vulnerable to COVID-19-related stressors. The long-standing added effects of social disparities can further compound this problem. To our knowledge, this is the first study to examine associations between COVID-19-related stressors and parental mental health, resilience, and parent-child engagement activities among parents of very young children residing in the Bronx, NY, a community that has long been burdened by inequities related to health, housing, income, race/ethnicity and environmental exposures. This community suffered heavily as an epicenter of the COVID-19 pandemic and continues to experience its aftermath.

In the Bronx MomBa Health Study participants, we observed that our study participants experienced comparable COVID-19-related exposures/events when compared to the national CEFIS validation study (25). Parents also reported on hardships they encountered qualitatively when answering an open-ended question on the effects of the pandemic, highlighting the challenges faced and coping strategies used by parents. Importantly, as has previously been reported (36, 37), despite high exposures to COVID-19-related events, some of our study participants reflected on the positive aspects of the pandemic including more family time and engaging children in creative indoor activities. Some of the parents also valued the opportunity of watching their children grow and achieve milestones, an experience that was made possible because of the pandemic lockdown and restricted activities after the lockdown was discontinued.

In our cohort, we have demonstrated a positive association between exposures to COVID-19-related events and higher levels of parental psychological distress among parents of very young children. There also was a positive association between COVID-19-related events and parent-reported psychological distress in their young children. This finding is consistent with prior reports of emotional/psychological distress in children during the COVID-19 pandemic, including clinginess, irritability, distraction, anxiety symptoms, and disturbed sleep and dietary habits (38, 39). However, most of these studies have focused on school aged children and adolescents. Although there was an inverse relationship between resilience and parent psychological distress, parental resilience did not protect them or their children from psychological distress secondary to COVID-19-related exposures. Of note, a recent meta-analysis of mental health symptoms of mothers with children under 5 years of age during the COVID-19 pandemic reported prevalence estimates of 26.9% and 41.9% for clinically significant depression and anxiety symptoms, respectively (40).

As expected, there was an inverse relationship between parental resilience and overall psychological distress as measured by the K10 survey. However, we observed that parent resilience did not protect against COVID-19-related psychological distress, suggesting that the pandemic-related additional stress may have overwhelmed their coping abilities. Similarly, parent resilience was unable to buffer the psychological distress experienced by their children. Not surprisingly, we observed that COVID-19-related psychological distress in parents and their children was highly correlated. Previous studies that have shown a strong association between the mental health of parents and their children (41, 42). More recent studies have shown that parent psychological distress, depression and anxiety during the pandemic adversely affects their children's behavior and emotional regulation and is associated with psychological symptoms in children and adolescents (43–45). It is important to note that unlike other studies, our cohort comprises of parents of very young children less than 2 years of age and therefore, child psychological distress was based on parent report rather than directly measured from the children themselves.

In our cohort, housing insecurity, but not food insecurity, was associated with increased psychological distress, highlighting the importance of identifying families with risk factors for early intervention. As expected, food insecurity was associated with increased use of food assistance programs. It is important to note that families with greater unmet food needs are more likely to apply for food assistance programs (46). Importantly, parents who utilized food assistance programs had higher psychological distress; however, this association was not statistically significant. Although previous studies have shown decreased psychological stress with the use of food assistance programs (47), more recent studies during the pandemic have shown that COVID-19 exacerbated economic hardship, food insecurity and psychological distress in urban families using food assistance programs (48).

Previous studies have shown that maternal psychological distress is associated with fewer positive parenting practices during early childhood (11–13). However, we did not observe any association between psychological distress or resilience levels and positive parenting practices in parents of very young children. Positive and consistent parenting behaviors after life stressors have been shown to improve child mental health outcomes. More recently, positive parenting practices have been shown to effectively buffer negative effects of COVID-19-related stress on school-aged children (23). Furthermore, more engagement of preschool children in family routines was associated with lower rates of child depressive and conduct problems during the pandemic (49).

Disparities in parenting practices among children under 5 years between economically advantaged and disadvantaged parents have previously been reported (16, 18–20). Consistent with these data, we observed that sleep routines and more frequent reading were associated with higher parental education, and child sex (female) was associated with parental storytelling. Interestingly, we observed that family dinners were associated with non-Hispanic ethnicity. This contrasts with prior research which shows a commitment to strong family ties amongst Hispanics. According to the US Census Bureau data, in 2018, 87% of Hispanic parents shared frequent meals with their children compared to 83% of non-Hispanic parents (50). Higher levels of exposures to COVID-19-related events were not associated with parent-child engagement activities, likely highlighting the complexity of multi-level factors existing before the pandemic and arising post-pandemic in our population that contribute to parent-child dynamics. In comparison to the pre-pandemic period, Bates et al. reported families engaged in fewer routines during the pandemic (51).

Strengths of our study include the racial and ethnic diversity of our participants. Additionally, our participants are parents of very young children, a group that is at greater risk of experiencing short- and long-term adverse effects related to the pandemic. Study limitations include a relatively small sample size which limited our ability to perform mediation or moderation analyses. Additional limitations include cross-sectional study design, inherent limitations of questionnaire data such as parent recall bias, and the use of one question vs. a longer scale for the parent-child engagement activities which will not capture as many dimensions of behavior or activity. Additionally, parents likely answered the COVID-19 Exposure and Family Impact Scales questions while considering all their children, and not just their recent newborn. As every family unit differs in structure, varied experiences may introduce variability in the COVID-19 Exposure and Family Impact Scales responses (i.e., “schools were closed” may only be applicable if an older sibling attended school). It is important to note that the COVID-19 Exposure and Family Impact Scales performed well in our cohort and showed high internal consistency. While adapting questionnaires to unique study populations can be beneficial, utilizing standardized and validated questionnaires like COVID-19 Exposure and Family Impact Scales is important to enhance generalizability of research findings.

The COVID-19 pandemic can be conceptualized as a traumatic event that may lead to enduring stress effects during vulnerable developmental periods such as the first 1,000 days of life. It is therefore critical to further investigate the effects of the pandemic and its consequences on child and family well-being. Identifying protective factors to support families with young children during high stress events may help mitigate some of the COVID-19 pandemic's negative effects. This study lays the foundation for future investigation to examine the longitudinal effects of the COVID-19 pandemic on parental mental health and resilience. Future studies should focus on how positive parent-child engagement activities impact the mental and emotional health of both parents and their children during high stress times.

## Data Availability

The raw data supporting the conclusions of this article will be made available by the authors, without undue reservation.
