# Corrigendum: Association of parent-child interactions with parental psychological distress and resilience during the COVID-19 pandemic

**DOI:** 10.3389/fped.2023.1245866

**Published:** 2023-08-21

**Authors:** Mana Mann, David Harary, Shirley Louis, Tao Wang, Karen Bonuck, Carmen R. Isasi, Maureen J. Charron, Mamta Fuloria

**Affiliations:** ^1^ Department of Pediatrics, Flushing Hospital Medical Center, Queens, NY, United States; ^2^ Department of Pediatrics, Division of Neonatology, Children’s Hospital at Montefiore, Albert Einstein College of Medicine, Bronx, NY, United States; ^3^ Department of Pediatrics, Division of Neonatology, Children’s Hospital of Michigan, Detroit, MI, United States; ^4^ Department of Epidemiology and Population Health, Albert Einstein College of Medicine, Bronx, NY, United States; ^5^ Department of Family and Social Medicine, Albert Einstein College of Medicine, Bronx, NY, United States; ^6^ Department of Obstetrics & Gynecology and Women’s Health, Albert Einstein College of Medicine, Bronx, NY, United States; ^7^ Department of Pediatrics, Division of Adolescent Medicine, Albert Einstein College of Medicine, Bronx, NY, United States; ^8^ Department of Biochemistry, Albert Einstein College of Medicine, Bronx, NY, United States; ^9^ Department of Medicine, Division of Endocrinology, Albert Einstein College of Medicine, Bronx, NY, United States

**Keywords:** COVID-19 pandemic, Bronx mother baby health study, parental resilience, psychological distress, parent-child interactions

A Corrigendum on Association of parent-child interactions with parental psychological distress and resilience during the COVID-19 pandemic By Mann M, Harary D, Louis S, Wang T, Bonuck K, Isasi CR, Charron MJ and Fuloria M (2023). Front. Pediatr. 11:1150216. doi: 10.3389/fped.2023.1150216

In the published article, there was an error regarding the affiliations for Karen Bonuck. Affiliation 1 “Department of Pediatrics, Flushing Hospital Medical Center, Queens, NY, United States” was assigned to her by error.

**Text Correction**

In the published article, there was an error in the symbols used.

A correction has been made to **Methods**, *Parent-child engagement activities*. This sentence previously stated:

“Activities were categorized and analyzed independently as present if children and caregivers engaged in: (1) family dinners ≥5 days/week, (2) sleep routines ≥5 days/week, (3) reading ≥3 times/week, (4) storytelling >3 = times/week, (5) singing >3 = times/week, or (6) playing >3 = times/week (17).”

The corrected sentence appears below:

“Activities were categorized and analyzed independently as present if children and caregivers engaged in: (1) family dinners ≥5 days/week, (2) sleep routines ≥5 days/week, (3) reading ≥3 times/week, (4) storytelling ≥3 times/week, (5) singing  ≥3 times/week, or (6) playing  ≥3 times/week (17).”

The authors apologize for these errors and state that this does not change the scientific conclusions of the article in any way. The original article has been updated.

